# Burden of prostate cancer in the Middle East: A comparative analysis based on global cancer observatory data

**DOI:** 10.1002/cam4.6689

**Published:** 2023-11-06

**Authors:** Garrett Kearney, Ming‐Hui Chen, Layth Mula‐Hussain, Mac Skelton, Mehmet Fuat Eren, Peter F. Orio, Paul L. Nguyen, Anthony V. D'Amico, Mutlay Sayan

**Affiliations:** ^1^ Department of Radiation Oncology Brigham and Women's Hospital and Dana Farber Cancer Institute, Harvard Medical School Boston Massachusetts USA; ^2^ Department of Statistics University of Connecticut Storrs Connecticut USA; ^3^ Faculty of Medicine Dalhousie University Halifax Nova Scotia Canada; ^4^ Institute of Regional and International Studies American University of Iraq Sulaimani Iraq; ^5^ Department of Radiation Oncology Marmara University Istanbul Pendik Education and Research Hospital Istanbul Turkey

**Keywords:** clinical observations, epidemiology and prevention, prostate cancer, risk assessment

## Abstract

**Background:**

Prostate cancer represents a significant global health issue, yet our understanding of its impact in the Middle East remains limited. This study aimed to assess the incidence and mortality of prostate cancer in the Middle East, and compare these rates to those in Europe and North America.

**Materials and Methods:**

We utilized the 2020 Global Cancer Observatory data, compiling incidence and mortality rates of prostate cancer in 20 Middle Eastern countries. We calculated mortality‐to‐incidence ratios (MIR) and compared the age‐standardized incidence rate (ASIR) and MIR between the Middle East and the combined regions of North America and Europe. The countries were further stratified based on the Human Development Index (HDI) and income level for additional analysis.

**Results:**

In 2020, the Middle East documented an estimated 51,649 new prostate cancer diagnoses, accounting for 3.7% of global cases. Despite a significantly lower ASIR in the Middle East compared with Europe and North America (10.50 vs. 21.50, *p* = 0.0087), the region had a higher MIR (12.35 vs. 3.00, *p* = 0.0476). When stratified based on HDI or income levels, there was no significant difference in MIRs; however, a significant trend of increasing MIR with decreasing HDI (*p* = 0.028) and income levels (*p* = 0.016) was observed.

**Conclusions:**

Despite a lower incidence, our analysis showed a significantly higher MIR for prostate cancer in the Middle East compared with Europe and North America. These findings underscore the unique challenges posed by prostate cancer in the Middle East and emphasize the necessity of tailored strategies to address this pressing public health issue.

## 1 INTRODUCTION

Prostate cancer is recognized as one of the most prevalent forms of cancer affecting men worldwide.^1^ As a complex disease, it presents a significant health burden that varies across geographic regions, age groups, and racial/ethnic populations.^2^ The nature of prostate cancer is such that its diagnosis and prognosis are influenced by a multitude of factors including genetic predisposition, lifestyle factors, and healthcare access.^3^, ^4^ Despite the considerable advancements in understanding its etiology and advancements in treatments, prostate cancer remains a leading cause of cancer‐related morbidity and mortality among men globally.

In contrast to regions like Europe and North America, where extensive research has been conducted to elucidate the disease's characteristics and its impact on populations, the status of prostate cancer in the Middle East is significantly less understood.^5^ A multitude of factors contribute to this knowledge gap including limited resources for comprehensive cancer registries, cultural barriers to healthcare access, and varied healthcare systems across the countries in this region.^6^, ^7^, ^8^ The available small institutional data suggest a consistent increase in the incidence of prostate cancer in this region over the last decade.^9^ This increasing trend, combined with a rapidly aging population, has raised serious concerns about the future healthcare burden and costs that the region will have to bear. The lack of robust epidemiological data in this setting poses substantial challenges in developing effective strategies for prostate cancer prevention, early detection, and management in the Middle East.

Despite the high prevalence and significant health impact of prostate cancer, there is an inadequate understanding of the disease's epidemiology in the Middle East, which consequently affects the formulation of health policies and strategies. Utilizing data from the Global Cancer Observatory (GCO), this study aims to shed light on the status of prostate cancer in the Middle East, thereby providing valuable insights that could guide the development of more effective, region‐specific health policies and interventions.

## 2 MATERIALS AND METHODS

The present study primarily utilizes data from the GCO 2020, an internationally recognized database that serves as a comprehensive source of cancer statistics worldwide.^10^ The GCO estimates are derived from the most recent data available to the International Agency for Research on Cancer (IARC), often in collaboration with population‐based cancer registries and the World Health Organization (WHO). It is important to note that these data sources are continually improving in terms of the quality and availability of data, which may lead to changes in methodology over time. As a result, the estimates in the GCO may not always be directly comparable with those published in previous versions of the dataset, such as the 2018 version. However, the GCO provides valuable insights into cancer epidemiology, and we employed these estimates while acknowledging the potential variations. The decision to use data from 2020 was based on the availability of the most current and up‐to‐date dataset.

The incidence and mortality rates of prostate cancer were compiled from the GCO database. A key metric employed in our analysis was the mortality‐to‐incidence ratio (MIR), a valuable epidemiological indicator often used to gauge the effectiveness of cancer management in populations. MIR was calculated by dividing the number of mortality cases by the number of incident cases. Additionally, we utilized the age‐standardized incidence rate (ASIR) and the age‐standardized mortality rate (ASMR), which were precalculated by the GCO, thus accounting for the age distribution differences in the populations.

The geographical focus of our analysis encompassed countries in the Middle East, namely, Turkey, Iraq, Iran, Syria, Yemen, Morocco, Algeria, Egypt, Jordan, Lebanon, Libya, Tunisia, Bahrain, Israel, Kuwait, Oman, Qatar, Saudi Arabia, United Arab Emirates, and territories such as the Gaza Strip and the West Bank. To elucidate the prostate cancer landscape across different sociodemographic strata, we compared the ASIR, ASMR, and MIR between the Middle East and combined regions of North America and Europe, using two‐sided exact Wilcoxon non‐parametric tests. North America and Europe were selected for comparison due to their well‐established healthcare systems, extensive cancer research, high incidence of prostate cancer, and the availability of high‐quality cancer data, enabling robust comparative analyses. Additionally, to conduct a deeper examination of the incidence and MIR of prostate cancer within the Middle East, countries were subgrouped based on the Human Development Index (low, medium, high, and very high defined by the United Nations Development Program^11^) as well as by income categorizations (low, lower middle, upper middle, and high income as determined by the World Bank^12^) (Table A1). HDI categorization offers insights into the broader development factors, encompassing education and life expectancy. Income categorization specifically addresses resource availability for healthcare, emphasizing the economic aspects of healthcare provision. For statistical analysis, the differences in ASIR, ASMR, and MIR among the subgroups were assessed using an Analysis of Variance (ANOVA) test. Since there was only a single observation in the “Low” HDI group, it was combined with the “Medium” HDI category for the statistical analysis. In addition to the ANOVA test, a linear trend analysis was also employed. This was used to discern any potential trends in ASIR, ASMR, and MIR across the ordered categories of HDI and income levels, thereby providing a more granular view of the data even when overarching group differences did not reach statistical significance. All statistical analyses were performed using SPSS, version 28. This study met the exception criteria from review by the Dana‐Farber Cancer Institute (DFCI) Institutional Review Board (IRB).

## 3 RESULTS

In the year 2020, it was estimated that the Middle Eastern countries collectively reported 51,649 new prostate cancer cases, comprising 3.7% of the global incidence. Within the region, the mortality for prostate cancer was 18,789.

A comparative analysis of the ASIR of prostate cancer between the Middle East and the combined regions of Europe and North America yielded significant disparities (Table 1). The ASIR in the Middle East was 10.50, considerably lower than the rate of 21.50 observed in Europe and North America (*p* = 0.0087). Conversely, the MIR in the Middle East was significantly higher than that reported in Europe and North America (12.35 vs. 3.00, *p* = 0.0476). This indicates that in the Middle Eastern region, there is a proportionally higher mortality rate among individuals diagnosed with prostate cancer compared with Europe and North America.

**TABLE 1:** Comparative analysis of prostate cancer epidemiology between the Middle East and the combined regions of Europe and North America.

	Middle East	Europe and North America	*p*
ASIR	10.50	21.50	0.0087
ASMR	10.95	17.00	0.2554
MIR	12.35	3.00	0.0476

*Note*: Age‐standardized rates are per 100,000 person‐years, calculated using a standard population. *P*‐values were derived from two‐sided exact Wilcoxon non‐parametric tests.

Abbreviations: ASIR, age‐standardized incidence rate; ASMR, age‐standardized mortality rate; MIR, mortality‐to‐incidence ratio.

In the stratified analysis based on the HDI, no significant difference was observed in the MIR of prostate cancer (Table 2). However, a significant association between income level and MIR on linear trend analysis was observed (*p* = 0.028), with the MIR increasing from 0.30 in very high HDI countries to 0.45 in low‐to‐medium HDI countries. Similarly, when stratifying countries based on income level, no significant difference was found in terms of the prostate cancer incidence and MIR. Nevertheless, a linear trend analysis revealed a significant association between income level and MIR (*p* = 0.016), with the MIR increasing from 0.29 in very high‐income countries to 0.50 in low‐income countries. These findings indicate that despite the absence of a significant overarching group difference, there is a clear trend of increasing MIR with decreasing HDI or income levels.

**TABLE 2:** Prostate cancer incidence and mortality rates in Middle Eastern countries stratified by HDI and income level.

	HDI			*p*
	Low‐to‐medium	High	Very high	
ASIR	14.58	18.59	23.35	0.473
ASMR	6.55	7.81	8.11	0.853
MIR	0.45	0.42	0.30	0.072

	Income level				*p*
	Low	Lower middle	Upper middle	High	
ASIR	10.90	19.70	22.45	20.61	0.742
ASMR	4.60	8.51	7.80	7.65	0.772
MIR	0.50	0.42	0.36	0.29	0.138

*Note*: Age‐standardized rates are per 100,000 person‐years, calculated using a standard population. *P*‐values were derived from ANOVA (analysis of variance) tests.

Abbreviations: HDI, Human Development Index; ASIR, age‐standardized incidence rate; ASMR, age‐standardized mortality rate; MIR, mortality‐to‐incidence ratio.

## 4 DISCUSSION

In this study, we conducted a comprehensive analysis of the incidence and mortality of prostate cancer in the Middle East using the 2020 GCO data. We found a significantly lower ASIR but a higher MIR for prostate cancer in the Middle East when compared to Europe and North America. In addition, there was a significant trend when countries stratified based on HDI and income levels, where countries with low‐to‐medium HDI or low‐income level had a higher MIR.

Our study's findings are particularly consequential as they underscore the critical and escalating public health challenge of prostate cancer in the Middle East. Despite observing lower incidence rates, the region exhibits a disproportionately higher MIR, which signifies a greater mortality rate among individuals diagnosed with prostate cancer. This pattern strongly suggests potential disparities in cancer care and outcomes. The significance of these findings is amplified by the scarcity of existing data on the epidemiology of prostate cancer in the Middle East, which highlights the importance of our study in filling this knowledge gap. These results warrant an immediate reevaluation and enhancement of the prevailing cancer care infrastructure in the region, including aspects of early detection, access to health care, and the quality of available treatments. This encompasses broad domains from strengthening screening programs, fostering healthcare access regardless of socio‐economic status, to ensuring the provision of up‐to‐date, high‐quality treatment regimens.

Our conclusions find support in prior research conducted in this area. Notably, a significant negative correlation between the HDI and the MIR across all cancer groups, including prostate cancer, has been documented.^13^, ^14^, ^15^, ^16^, ^17^ These findings corroborate our observations concerning the linkage between the burden of prostate cancer and socioeconomic factors. Furthermore, research by Sharma et al. aligns with our results, demonstrating that prostate cancer, despite being the leading malignancy among males in Africa, is associated with worse mortality rates and a higher MIR compared with other continents.^17^ This study also demonstrated that the HDI accounted for 75% of the variation in overall 5‐year cancer survival, emphasizing the influence of socio‐economic conditions on cancer outcomes. Therefore, our study, along with these corroborating research efforts, highlights the pressing need to address the burden of prostate cancer in the Middle East.

A confluence of unique circumstances and socio‐economic conditions within the Middle East could elucidate the paradox of higher prostate cancer mortality despite lower incidence rates in the region. One primary factor is the alarming rate of advanced prostate cancer diagnoses.^7^, ^9^, ^18^ While the incidence of prostate cancer is increasing, data on the stage at diagnosis and treatment outcomes remain scarce, potentially leading to increased mortality rates. Concurrently, lower incidence rates might be attributed to biological features of male populations in the Arab World, as suggested by several studies.^5^, ^19^, ^20^ Access to health care, an important determinant of cancer outcomes, is unfortunately limited or negatively impacted by conflict and displacement in many parts of the Middle East,^21^, ^22^, ^23^ which could result in delayed diagnosis and consequently, higher mortality rates. Lastly, cultural and societal barriers, including stigma around cancer and lack of awareness about the importance of early detection, can delay diagnosis and lead to higher rates of advanced disease at presentation.^8^, ^24^ Taken together, these factors elucidate the complexity of addressing the burden of prostate cancer in the Middle East, necessitating tailored, region‐specific solutions that account for these unique challenges.

Addressing the higher MIR of prostate cancer in the Middle East calls for a strategic approach to improve access to healthcare and screening programs. This requires increasing public awareness about the disease, its symptoms, and the importance of early detection, which can promote timely diagnosis and improve treatment outcomes. The implementation of targeted screening programs across the region is critical in this regard. Simultaneously, enhancing access to high‐quality healthcare, especially specialist multidisciplinary management, can dramatically improve patient outcomes. Investments in research are crucial to further our understanding of the reasons behind the observed higher MIR of prostate cancer in the Middle East. This research can aid in the identification and implementation of effective strategies to address this issue. Lastly, collaboration with international organizations can provide necessary resources and expertise to bolster local efforts in improving access to healthcare and screening programs. However, it is important to recognize that the decision to advocate for screening asymptomatic individuals solely based on PSA test results is complex, given the test's limitations in terms of false positives and overdiagnosis. Therefore, it is imperative that public awareness campaigns and healthcare guidance include a balanced discussion of the potential benefits and risks associated with PSA screening. This approach ensures that patients can make informed decisions about prostate cancer screening based on their individual health profiles and preferences.

In addition to our focus on ASIR and MIR, it is essential to discuss ASMR in the context of our study. We found that ASMR was not significantly difference between the Middle East and the combined regions of Europe and North America. ASMR reflects the probability of death from prostate cancer among individuals diagnosed with the disease and is influenced by several factors, including the absolute number of cases, treatment effectiveness, healthcare infrastructure, access to care, demographic factors, and age distribution. While the Middle East displayed a higher MIR, indicating a greater proportion of prostate cancer diagnoses resulting in mortality, the absence of a difference in ASMR suggests a nuanced interplay of these factors. Further research is warranted to explore the contributing factors to this similarity in ASMR between regions and to develop targeted interventions that address disparities in cancer care and outcomes.

While our study provides valuable insights into prostate cancer epidemiology in the Middle East and employs widely recognized epidemiological indicators for meaningful global comparisons, it is important to consider the following limitations. Firstly, it is important to note that the GCO data, while comprehensive, are retrospective in nature and rely on data available to the IARC, often in collaboration with population‐based cancer registries and the WHO. The accuracy and comprehensiveness of recorded data can vary considerably among different Middle Eastern countries, potentially leading to variations in the reported incidence and mortality rates of prostate cancer. Furthermore, as the data sources are continually improving in terms of quality and availability, the estimates in the GCO may not always be directly comparable with those published in previous versions of the dataset. Secondly, the COVID‐19 pandemic introduced unique challenges to the assessment of cancer incidence and mortality rates. Our study did not account for the specific impact of COVID‐19 on cancer rates, as the GCO data we utilized were not designed to capture this unprecedented situation. Finally, due to the nature of available data, we were unable to incorporate other confounding factors, such as genetic predispositions or lifestyle variables, that might influence the incidence and mortality rates of prostate cancer. Furthermore, we acknowledge the methodological limitation of statistical analysis used in our study to compare rates, which traditionally is suboptimal due to the specific statistical nature of rate data. The lack of available exposure data or the population at risk from the GCO database constrained our ability to utilize more precise models to yield incidence rate ratios and offer a potentially more robust analysis.

## 5 CONCLUSION

This study sheds light on the significant MIR of prostate cancer in the Middle East despite its lower incidence, highlighting the critical need for improved healthcare strategies and infrastructure. It emphasizes the importance of strengthening cancer registries, enhancing early detection programs, and ensuring equitable access to quality care. While more detailed and inclusive research is required to further our understanding, this study serves as a step forward in acknowledging and addressing the public health challenge that prostate cancer poses in the Middle East.

## AUTHOR CONTRIBUTIONS

**Garrett Kearney:** Conceptualization (equal); data curation (equal); methodology (equal); project administration (equal); writing – original draft (equal); writing – review and editing (equal). **Ming‐Hui Chen:** Conceptualization (equal); data curation (equal); formal analysis (equal); methodology (equal). **Layth Mula‐Hussain:** Conceptualization (equal); methodology (equal); writing – review and editing (equal). **Mac Skelton:** Conceptualization (equal); methodology (equal); writing – review and editing (equal). **Mehmet Fuat Eren:** Conceptualization (equal); methodology (equal); writing – review and editing (equal). **Peter F. Orio:** Conceptualization (equal); methodology (equal); writing – review and editing (equal). **Paul L. Nguyen:** Conceptualization (equal); methodology (equal); writing – review and editing (equal). **Anthony D'Amico:** Conceptualization (equal); methodology (equal); writing – review and editing (equal). **Mutlay Sayan:** Conceptualization (equal); data curation (equal); formal analysis (equal); methodology (equal); writing – original draft (equal); writing – review and editing (equal).

## CONFLICT OF INTEREST STATEMENT

The authors declare no conflict of interest.

## CONDENSED ABSTRACT

Despite a lower incidence, the Middle East carries a significantly higher MIR for prostate cancer compared with Europe and North America, emphasizing the need for region‐specific healthcare strategies.

**TABLE A1:** Prostate cancer epidemiology in Middle Eastern countries stratified by HDI and income level.

	Human Development Index		
	Low‐to‐medium	High	Very high
Countries	Yemen Syria Iraq Morocco	Algeria Egypt Iran Jordan Lebanon Libya Tunisia Gaza and West Bank	Bahrain Israel Kuwait Oman Qatar Saudi Arabia Turkey United Arab Emirates
ASIR			
Median	15.95	17.0	16.7
Range	2.8–23.6	13.9–28.5	7.0–56.1
IQR	10.38, 20.15	16.48, 19.25	13.38, 26.45
ASMR			
Median	6.35	7.65	6.3
Range	1.7–11.8	5.3–10.0	2.5–22.5
IQR	4.33, 8.58	6.60, 9.48	4.73, 7.85
MIR			
Median	0.41	0.46	0.26
Range	0.37–0.61	0.31–0.48	0.16–0.69
IQR	0.39, 0.47	0.35, 0.47	0.20–0.32

	Income level			
	Low	Lower middle	Upper middle	High
Countries	Yemen Syria	Morocco Algeria Egypt Gaza and West Bank Iran Lebanon Tunisia	Iraq Jordan Libya Turkey	Bahrain Israel Kuwait Oman Qatar Saudi Arabia United Arab Emirates
ASIR				
Median	10.9	17.0	17.2	13.8
Range	2.8–19	13.9–28.5	12.9–42.5	7.0–56.1
IQR	6.85, 14.95	16.85, 22.4	15.08, 24.58	13.35, 20.35
ASMR				
Median	4.6	7.9	7.35	6.0
Range	1.7–7.5	6.0–11.8	5.2–11.3	2.5–22.5
IQR	3.15, 6.05	7.10, 9.85	5.25, 9.88	4.65, 6.65
MIR				
Median	0.51	0.45	0.34	0.24
Range	0.39–0.62	0.33–0.48	0.28–0.47	0.16–0.63
IQR	0.45, 0.56	0.39, 0.47	0.30, 0.40	0.19, 0.34

## Data Availability

This study data that support the findings are available on request from the corresponding author.

## References

[1] Pernar CH , Ebot EM , Wilson KM , Mucci LA . The epidemiology of prostate cancer. Cold Spring Harb Perspect Med. 2018;8(12):a030361. 29311132 10.1101/cshperspect.a030361 PMC6280714

[2] Park SY , Haiman CA , Cheng I , et al. Racial/ethnic differences in lifestyle‐related factors and prostate cancer risk: the multiethnic cohort study. Cancer Causes Control. 2015;26:1507‐1515. 26243447 10.1007/s10552-015-0644-y PMC4567936

[3] Kazmi N , Haycock PC , Tsilidis KK , et al. Appraising causal relationships of dietary, nutritional and physical‐activity exposures with overall and aggressive prostate cancer: two‐sample mendelian‐randomization study based on 79 148 prostate‐cancer cases and 61106 controls. Int J Epidemiol. 2019;49:587‐596. 10.1093/ije/dyz235 31802111

[4] Layne TM , Graubard BI , Ma X , Mayne ST , Albanes D . Prostate cancer risk factors in black and white men in the NIH‐AARP diet and health study. Prostate Cancer Prostatic Dis. 2018;22:91‐100. 30108373 10.1038/s41391-018-0070-9 PMC6676904

[5] Ali AH , Awada H , Nassereldine H , et al. Prostate cancer in the Arab world: bibliometric review and research priority recommendations. Arab J Urol. 2022;20(2):81‐87. 35530565 10.1080/2090598X.2021.2024984 PMC9067956

[6] Saad M , Alip A , Lim J , et al. Management of advanced prostate cancer in a middle‐income country: real‐world consideration of the advanced prostate cancer consensus conference 2017. BJU Int. 2019;124:373‐382. 31077523 10.1111/bju.14807 PMC6851975

[7] Eren MF , Kilic SS , Eren AA , et al. Radiation therapy for prostate cancer in Syrian refugees: facing the need for change. Front Public Health. 2023;11:1172864. 37325331 10.3389/fpubh.2023.1172864 PMC10264678

[8] Al‐Abdin OZ , Al‐Beeshi IZ . Prostate cancer in the Arab population. An verview. Saudi Med J. 2018;39(5):453‐458. 29738003 10.15537/smj.2018.5.21986 PMC6118189

[9] Daher M , Telvizian T , Dagher C , et al. High rates of advanced prostate cancer in the Middle East: analysis from a tertiary care center. Urol Ann. 2021;13(4):418‐423. 34759656 10.4103/UA.UA_47_20 PMC8525480

[10] Global Cancer Observatory . The International Agency for Research on Cancer. World Health Organization. Accessed June 20, 2023. https://gco.iarc.fr/

[11] Human Development Insights . Human Development Reports. Accessed June 20, 2023. https://hdr.undp.org/data‐center/country‐insights#/ranks

[12] The World by Income . The World Bank. Accessed June 20, 2023. https://datatopics.worldbank.org/world‐development‐indicators/the‐world‐by‐income‐and‐region.html

[13] Pakzad R , Mohammadian‐Hafshejani A , Ghoncheh M , Pakzad I , Salehiniya H . The incidence and mortality of prostate cancer and its relationship with development in Asia. Prostate Int. 2015;3:135‐140. 26779461 10.1016/j.prnil.2015.09.001 PMC4685206

[14] Hassanipour‐Azgomi S , Mohammadian‐Hafshejani A , Ghoncheh M , Towhidi F , Jamehshorani S , Salehiniya H . Incidence and mortality of prostate cancer and their relationship with the human development index worldwide. Prostate Int. 2016;4:118‐124. 27689070 10.1016/j.prnil.2016.07.001 PMC5031898

[15] Soheylizad M , Khazaei S , Jenabi E , Delpisheh A , Veisani Y . The relationship between human development index and its components with thyroid cancer incidence and mortality: using the decomposition approach. Int J Endocrinol Metab. 2018;16(4):e65078. 30464773 10.5812/ijem.65078 PMC6218660

[16] Khazaei Z , Goodarzi E , Borhaninejad V , et al. The association between incidence and mortality of brain cancer and human development index (HDI): an ecological study. BMC Public Health. 2020;20(1):1696. 33183267 10.1186/s12889-020-09838-4 PMC7664078

[17] Sharma R , Aashima N , Nanda M , et al. Mapping cancer in Africa: a comprehensive and comparable characterization of 34 cancer types using estimates from GLOBOCAN 2020. Front Public Health. 2022;10:839835. 35548083 10.3389/fpubh.2022.839835 PMC9082420

[18] Abbasi‐Kangevari M , Moghaddam SS , Ghamari S‐H , et al. The burden of prostate cancer in North Africa and Middle East, 1990–2019: findings from the global burden of disease study. Front Oncol. 2022;12:961086. 36176394 10.3389/fonc.2022.961086 PMC9513750

[19] Kehinde EO , Mojiminiyi OA , Sheikh M , et al. Age‐specific reference levels of serum prostate‐specific antigen and prostate volume in healthy Arab men. Br J Urol. 2005;96:308‐312. 10.1111/j.1464-410X.2005.05620.x 16042719

[20] Arafa MA , Rabah DM . With increasing trends of prostate cancer in the Saudi Arabia and Arab world: should we start screening programs? World. J Clin Oncol. 2017;8:447‐449. 10.5306/wjco.v8.i6.447 PMC5740100 29291169

[21] Mukherji D , Youssef B , Dagher C , et al. Management of patients with high‐risk and advanced prostate cancer in the Middle East: resource‐stratified consensus recommendations. World J Urol. 2019;38:681‐693. 31297628 10.1007/s00345-019-02872-x PMC7064460

[22] El Saghir NS , Pérez S , de Celis E , Fares JE , Sullivan R . Cancer care for refugees and displaced populations: Middle East conflicts and global natural disasters. Am Soc Clin Oncol Educ Book. 2018;38:433‐440. 30231320 10.1200/EDBK_201365

[23] Skelton M , Alameddine R , Saifi O , et al. High‐cost cancer treatment across borders in conflict zones: experience of Iraqi patients in Lebanon. JCO Glob Oncol. 2020;6:59‐66. 32031440 10.1200/JGO.19.00281 PMC6998032

[24] Kizilkaya MC , Kilic SS , Bozkurt MA , et al. Breast cancer awareness among afghan refugee women in Turkey. EClinicalMedicine. 2022;49:101459. 35747185 10.1016/j.eclinm.2022.101459 PMC9168491

